# Sierra Nevada reference conditions: A dataset of contemporary reference sites and corresponding remote sensing-derived forest structure metrics for yellow pine and mixed-conifer forests

**DOI:** 10.1016/j.dib.2023.109807

**Published:** 2023-11-14

**Authors:** Caden P. Chamberlain, Gina R. Cova, Van R. Kane, C. Alina Cansler, Jonathan T. Kane, Bryce N. Bartl-Geller, Liz van Wagtendonk, Sean M.A. Jeronimo, Peter Stine, Malcolm P. North

**Affiliations:** a School of Environmental and Forest Sciences, University of Washington; Anderson Hall, 3715 W Stevens Way NE, Seattle, WA 98195, United States; b W.A. Franke College of Forestry & Conservation, University of Montana; 32 Campus Drive, Missoula, MT 59812, United States; c Resilient Forestry, Seattle, WA 98136, United States; d Climate & Wildfire Institute, CA, United States; e USDA Forest Service, PSW Research Station, Mammoth Lakes, CA 93546, United States

**Keywords:** Reference conditions, Sierra Nevada, Airborne lidar, California Forest Observatory, Forest structure

## Abstract

Contemporary reference sites in California's Sierra Nevada represent areas where a frequent, low-intensity fire regime – an integral ecological process in temperate dry forests – has been reintroduced after several decades of fire suppression. Produced by an intact fire regime, forest structural patterns in these sites are likely more resilient to future disturbances and climate, and thus can provide reference conditions to guide management and ecological research. In this paper, we present a set of 119 delineated contemporary reference sites in the Sierra Nevada yellow pine and mixed-conifer zone along with a suite of key remote sensing-derived forest structure metrics representing conditions within these sites. We also provide a set of summary figures for individual reference sites and sites grouped by dominant climate class. We identified restored frequent-fire landscapes using a combination of fire history, burn severity, management history, and forest type datasets and we delineated individual polygons using catchment basins, fire perimeters, and imagery. Reference sites ranged in size from 101-966 ha with a mean size of 240 ha. Where available (for 59 sites), we used airborne lidar datasets to characterize a suite of key forest structure metrics within reference sites. Across all 119 sites, we provide a set of forest structure metrics produced by the California Forest Observatory. Reference sites were categorized based on their dominant climate class to assist users in identifying the most climatically relevant reference conditions for their project or study area. We encourage the use of the reference sites and associated forest structure datasets for guiding ecologically focused forest management and research in the Sierra Nevada.

Specifications Table

**Table:** 

Subject	Ecology and Forestry
Specific subject area	Contemporary reference sites and forest structure
Data format	Shapefiles representing 1) contemporary reference sites, 2) Sierra Nevada climate classes, 3) land ownership, and 4) Sierra Nevada ecoregion. GeoTIFF rasters representing forest structure metrics derived from airborne lidar data (30- or 90-m resolution), California Forest Observatory metrics (10 m resolution), and USGS digital elevation model (10-m resolution). All spatial data is in California Teale Albers projection (EPSG: 3310). Spatial data provided in 1) ESRI ArcGIS Pro package, 2) raw zipped files, and 3) ESRI Web Map. PDFs with tables and figures representing forest structure metrics within contemporary reference sites.
Type of data	ESRI ArcGis Pro Package (.ppkg) ESRI Shapefile (.shp) GeoTIFF raster (.tif) PDF Document (.pdf)
Data collection	All downloaded spatial datasets (fire history, management history, forest type, catchment basins, and climate data, etc.) were from publicly available websites. Forest structure metrics were derived from six airborne lidar acquisitions: North/South Plumas National Forest, Eldorado National Forest, Tuolumne County, Yosemite National Park, and the Southern Sierra All Lands Restoration site. Lidar data was either 1) downloaded from the USGS bulk download site or 2) acquired via personal communication. We downloaded forest structure layers from the California Forest Observatory website. All lidar data was acquired during summer leaf-on months between 2018 and 2020. Acquisitions were all flown with at least 50% flightline overlap and resultant mean pulse density ranged from 12.6-28.0 pulses/m^2^. We used the USDA Forest Service's FUSION software to filter, normalize, and process point cloud data from all six acquisitions (more detail provided below).
Data source location	Datasets used to identify and delineate contemporary reference site polygons included: -CalFire's fire perimeters (https://frap.fire.ca.gov/mapping/gis-data/) -Knight et al. 2022 management history (https://doi.org/10.1073/pnas.2116264119) -USDA Forest Service FACTS database (https://data.fs.usda.gov/geodata/edw/datasets.php) -FVEG Landcover dataset (https://map.dfg.ca.gov/metadata/ds1327.html) -National Catchments dataset (https://www.epa.gov/waterdata/nhdplus-national-data) -ESRI world imagery (https://www.arcgis.com/home/item.html?id=c03a526d94704bfb839445e80de95495) Airborne lidar acquisitions used to derive forest structure metrics: -North Plumas National Forest (https://rockyweb.usgs.gov/vdelivery/Datasets/Staged/Elevation/LPC/Projects/CA_NoCAL_3DEP_Supp_Funding_2018_D18/CA_NoCAL_Wildfires_PlumasNF_B1_2018/) -South Plumas National Forest (https://rockyweb.usgs.gov/vdelivery/Datasets/Staged/Elevation/LPC/Projects/CA_NoCAL_3DEP_Supp_Funding_2018_D18/CA_NoCAL_Wildfires_PlumasNF_B2_2018/) -Eldorado National Forest (https://rockyweb.usgs.gov/vdelivery/Datasets/Staged/Elevation/LPC/Projects/CA_UpperSouthAmerican_Eldorado_2019_B19/CA_UpperSouthAmerican_Eldorado_2019/metadata/USGS_LPC_CA_UpperSouthAmerican_Eldorado_2019_B19_10SFG663633.xml) -Yosemite National Park (https://rockyweb.usgs.gov/vdelivery/Datasets/Staged/Elevation/LPC/Projects/CA_YosemiteNP_2019_D19/CA_YosemiteNP_2019) -SSARR (https://rockyweb.usgs.gov/vdelivery/Datasets/Staged/Elevation/LPC/Projects/CA_SouthernSierra_2020_B20/CA_SouthernSierra_1_2020/ -Tuolumne County (obtained via personal communication) California Forest Observatory forest structure metrics (https://forestobservatory.com/) Other base layer datasets: -EPA Level IV Sierra Nevada ecoregion (https://www.epa.gov/eco-research/ecoregion-download-files-state-region-9#pane-04) -USDA Forest Service boundaries (https://data.fs.usda.gov/geodata/edw/datasets.php) -DOI National Park Service boundaries (https://public-nps.opendata.arcgis.com/search?collection=Dataset&q=boundaries) -USGS 10 m digital elevation model (https://www.usgs.gov/tools/national-map-viewer)
Data accessibility	Repository name: Forest Service Research Data Archive Data identification number: doi.org/10.2737/RDS-2023-0027 Direct URL to data: https://www.fs.usda.gov/rds/archive/catalog/RDS-2023-0027
Related research article	Chamberlain, C.P., Cova, G.R., Cansler, C.A., North, M.P., Meyer, M.D., Jeronimo, S.M.A., Kane, V.R., 2023. Consistently heterogeneous structures observed at multiple spatial scales across fire-intact reference sites. For. Ecol. Manag. 550: 121478. https://doi.org/10.1016/j.foreco.2023.121478

## 1 Value of the Data

• Contemporary reference site polygons represent areas where a frequent, low-intensity fire regime has been reintroduced after more than a century of fire suppression. These sites, where pattern-process linkages are mostly intact, can be used to derive reference conditions, which are frequently required by natural resource managers and scientists.
• We provide a set of key forest structure datasets describing various components of the vertical and horizontal arrangement of trees, foliage, and other vegetation within the reference sites. Violin plots derived from the forest structure datasets depict the range and variability of reference conditions within sites.
• We provide several other spatial datasets to assist users in contextualizing the reference sites. Climatic and topographic metrics can be used to match reference site polygons (and metrics) with project areas of interest, and land ownership layers can provide insight about past, present, and future management practices in these sites.
• Scientists can use reference condition datasets to evaluate the effects of other management interventions or natural disturbances. We also encourage ongoing research and monitoring of these sites and their forest conditions as they continue to be affected by disturbances and climate change in years to come.
• We anticipate forest managers and planners will use forest structure datasets to assist in designing and evaluating ecologically centered management treatments in the Sierra Nevada ecoregion.

## 2 Data Description

We used fire history, burn severity, management history, and other remote sensing datasets to identify and delineate a set of contemporary reference sites in the yellow pine and mixed-conifer zone of the Sierra Nevada, California (see Methods). These sites represent areas with a mostly restored, frequent, and low-intensity fire regime [1]. We used airborne lidar data and compiled California Forest Observatory forest structure datasets to characterize structural conditions within the contemporary reference sites. All datasets have been archived on the Forest Service Research Data Archive [2].

We provide descriptions of the three primary datasets contained in the archive in Table 1, which include RDS_2023-0027_Data_PPKX.zip, RDS-2023-0027_Data_TIF_SHP_GPKG.zip, and SNCRS_Summaries.pdf.

**Table 1:** Data files shared in the Forest Service Research Data Archive.

File	Description
RDS_2023-0027_Data_PPKX.zip	ESRI ArcGIS Pro package including contemporary reference sites shapefile, Sierra Nevada ecoregion shapefile, Jeronimo et al. [3] climate classes shapefile, land ownership shapefile, USGS 10m resolution digital elevation model raster, 0.75 m lidar canopy height model raster, 0.75 m lidar canopy height model hillshade raster, 15 lidar-derived forest structure metric rasters, and 6 California Forest Observatory forest structure metric rasters
SNCRS_Summaries.pdf	PDFs with site descriptions, locations, and summary statistics for 1) all reference sites grouped by dominant climate class and 2) individual sites
RDS-2023-0027_Data_TIF_SHP_GPKG.zip	Zipped file containing all raw spatial data provided in the ArcGIS Pro package for non-ESRI users

The RDS_2023-0027_Data_PPKX.zip file contains an ESRI ArcGIS Pro package which contains shapefile and GeoTIFF raster datasets, symbolized for interpretability. The package contains:

• Contemporary reference site shapefiles
• Sierra Nevada ecoregion boundary
• Climate classes produced by Jeronimo et al. [3]
• National Forest Service and National Park Service land ownership boundaries
• USGS 10 m resolution digital elevation model
• 15 airborne lidar-derived forest structure raster layers
• 6 California Forest Observatory forest structure raster layers

The RDS-2023-0027_Data_TIF_SHP_GPKG.zip file contains all raw datasets listed above for non-ESRI users.

In addition to the spatial datasets, we provide a PDF document – SNCRS_Summaries.pdf – that includes summary statistics and figures for the contemporary reference sites. This document provides summaries for reference sites grouped by dominant climate class [3] as well as for individual reference sites. Summary pages include site descriptions (e.g., area, ownership, number of recent fires, etc.), geographic location, climatic context, and a set of violin plots showing the distribution of key forest structure metrics from airborne lidar (where available) and CFO datasets.

**Fig. 1:**
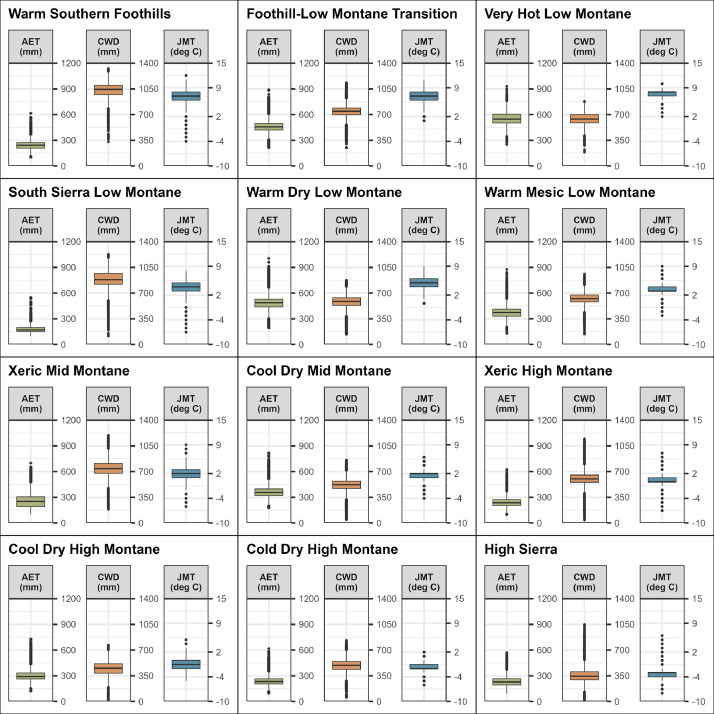
Distribution of actual evapotranspiration (AET), climatic water deficit (CWD), and January minimum temperature (JMT) for each of the 12 Jeronimo et al. [3] climate classes represented by the contemporary reference sites. Climate variables are 30-year averages for years 1981-2010 [9].

**Fig. 2:**
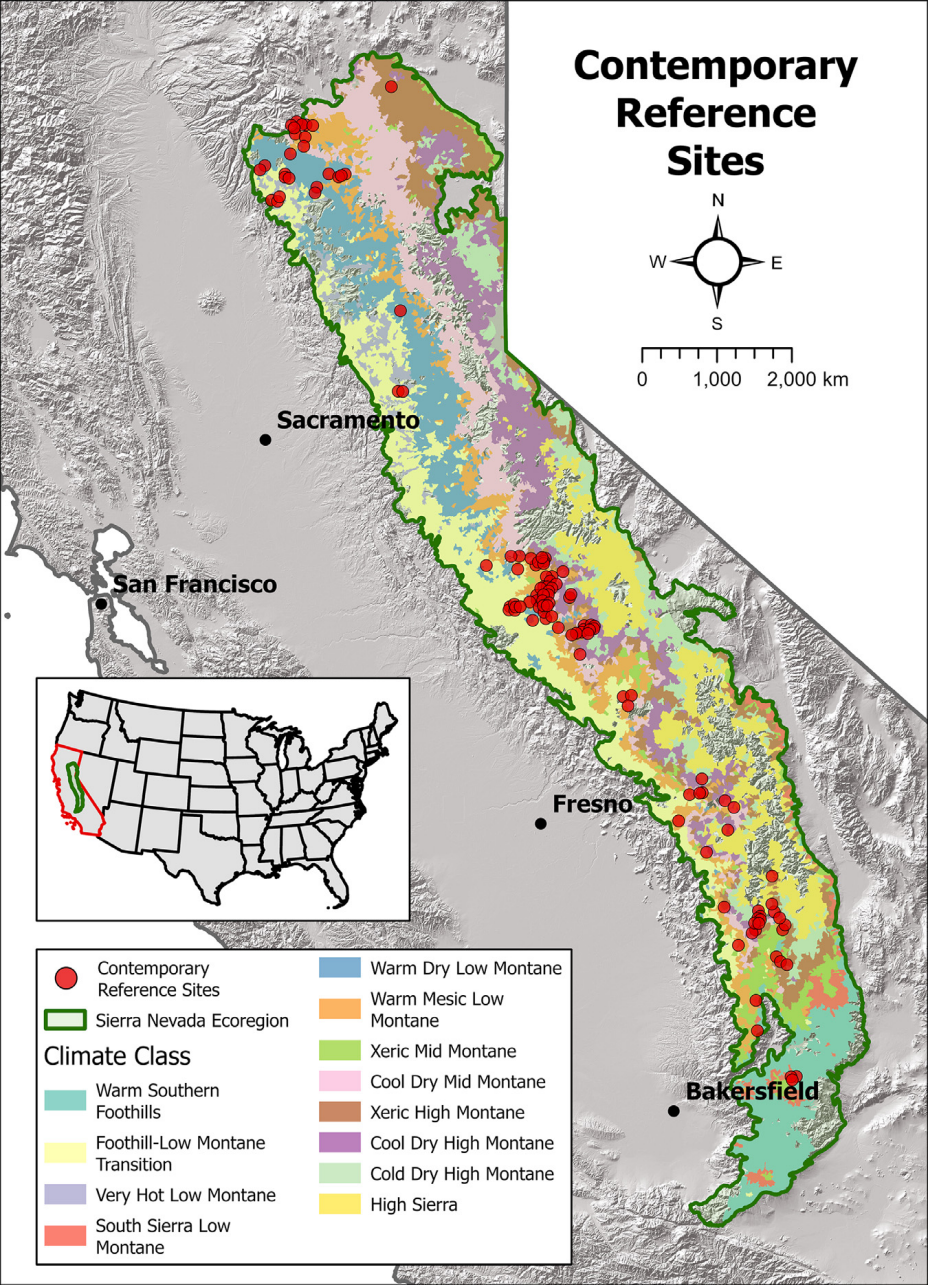
Map showing the location of the 119 contemporary reference sites and the distribution of the 12 Jeronimo et al. [3] climate classes within the Sierra Nevada ecoregion.

**Fig. 3:**
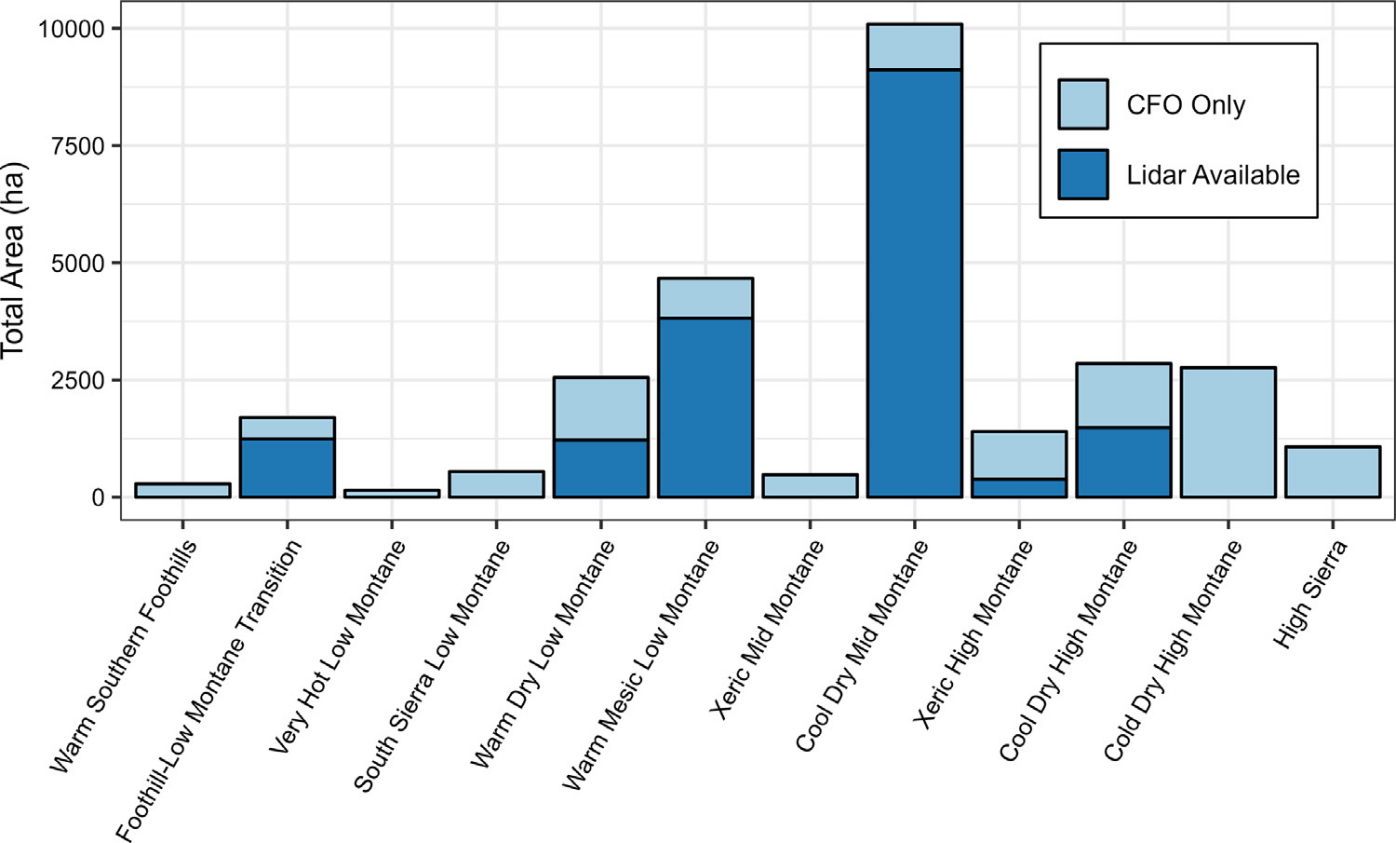
Total contemporary reference site area representing each of the 12 Jeronimo et al. [3] climate classes, with bars colored based on airborne lidar availability.

We include several figures and tables in this article to provide more context about the archived datasets. In Table 1 we provide descriptions of all datasets provided in the Forest Service Research Data Archive. In Table 2 we provide summary count and area statistics for the contemporary reference site polygons. In Fig. 1 we provide summary statistics for each of the 12 Jeronimo et al. [3] climate classes that were used to categorize the reference sites. In Fig. 2 we provide a map of the Sierra Nevada region and the location of the 119 contemporary reference sites in relation to the 12 climate classes. In Fig. 3 we show the total area of reference sites grouped by climate class to illustrate the extent to which different climate classes are represented. In Table 3 we provide information about the six airborne lidar acquisitions used to derive forest structure metrics within the reference sites. In Table 4 we provide a glossary of all forest structure metrics included in the archive with corresponding file names and metric descriptions. Lastly, in Figs. 5 and 6, we provide samples of the overview and individual reference site PDF pages provided in the SNCRS_Summaries.pdf document.

## 3 Experimental Design, Materials and Methods

Experimental design, materials, and methods used to identify and contextualize the contemporary reference sites and produce corresponding forest structure metrics are described in detail in the metadata files for the archived dataset [2]. Portions of the following section were pulled directly from the metadata document to ensure that descriptions between the two sources do not differ.

### 3.1 Identifying contemporary reference sites

We followed an approach developed by Jeronimo et al. [3] for identifying contemporary reference sites in the Sierra Nevada but used updated burn severity and management history datasets to produce a new dataset. Their approach involved 1) scoring rasters across the Sierra Nevada based on the degree to which each pixel represented a restored low-intensity, frequent fire regime, 2) selecting catchment polygons dominated by high scoring pixels, and 3) refining catchment boundaries using fire perimeter and imagery datasets. As described below, we implemented the same raster scoring criteria as Jeronimo et al. [3] but we included an additional criterion to ensure that only yellow pine and mixed-conifer forest types were analyzed. We defined the Sierra Nevada ecoregion as all area within the Environmental Protection Agency's Level IV Sierra Nevada Ecoregion, though we applied a 5-km buffer to this dataset to capture 3 contemporary reference sites that fell just north of the official Sierra Nevada boundary.

#### 3.1.1 Datasets

We used four primary datasets for raster scoring including fire history, burn severity, management history, and forest type. For delineating polygons, we used the national catchments dataset, fire perimeters, and ESRI imagery.

We used the CalFire Fire and Resource Assessment Program's (FRAP) Fire Perimeter dataset (https://frap.fire.ca.gov/mapping/gis-data/) to map all recent fire history. We retained records of all fires greater than 4 ha for years 1957-2020, including prescribed fires. The fire perimeter dataset was quality checked for duplicate records and topology errors [4]. For all fires that burned in or after 1985, we used the Google Earth Engine code developed by Parks et al. [5] to quantify and map burn severity as predicted Composite Burn Index (CBI) values. We generated bias corrected versions of our outputs to ensure high-severity patches were adequately mapped. All burn severity layers were classified into categories of unburned, low, moderate, and high severity using CBI thresholds recommended by Miller and Thode [6]. Prior to 1985, Landsat data was not available for modelling burn severity. Thus, for all pre-1985 fires in our dataset that intersected potential reference sites, we visually examined imagery and a lidar-derived canopy height layer (i.e., ‘dominant canopy height’, more detail below) for evidence of past stand-replacing fire and excluded all expected high-severity burn areas from our analyses.

For management history datasets we used 1) the Knight et al. [7] dataset for years 1985-2020 and 2) the USDA Forest Service FACTS database records for years prior to 1985 (https://data.fs.usda.gov/geodata/edw/datasets.php). The Knight et al. [7] dataset included all management history records from both the Forest Service FACTS database as well as the CalFire Timber Harvesting Plans (THP) database. These databases contain a variety of records including regeneration harvests, fuel treatments, prescribed burning, and administrative/monitoring tasks. Since we wished to produce a single record of management history representing “on-the-ground” treatments, we used tables from the Knight et al. [7] supplementary materials (e.g., Tables S4-S8) to classify and discard all management records representing monitoring or administrative tasks. Additionally, we excluded all treatments related to prescribed fire or broadcast burning since these records were accounted for in the FRAP fire history dataset. The Knight et al. [7] datasets only included records for 1985-2020, so we used the FACTS database for all management records prior to 1985. We used the same tables from the Knight et al. [7] supplementary materials to classify and discard monitoring, administrative, and prescribed burning records. Ultimately, we used the Knight et al. [7] and pre-1985 FACTS datasets to produce a final binary 30-m resolution raster representing treated versus non-treated pixels across the Sierra Nevada ecoregion.

We used the FVEG dataset (https://map.dfg.ca.gov/metadata/ds1327.html) to identify contemporary forest types representing the yellow pine and mixed-conifer zone of the western Sierra Nevada, as defined in Safford and Stevens [8]. Specifically, we included FVEG WHR codes for ‘Ponderosa Pine’, ‘Jeffrey Pine’, ‘Douglas-fir’, ‘Montane Hardwood-Conifer’, and ‘Sierran Mixed Conifer’. Using these five forest types we produced a binary 30-m resolution raster representing desired versus non-desired forest types.

#### 3.1.2 Delineating contemporary reference sites

We used the fire history, classified burn severity, management history, and forest type datasets described above to produce a scored 30-m resolution raster across the Sierra Nevada ecoregion. Following methods proposed in Jeronimo et al. [3], each pixel was assigned a point for each of the following true statements:

(1) At least 2 fires in the last 60 years
(2) At least one fire in the last 30 years
(3) At least one fire with moderate-severity effects
(4) No high-severity effects
(5) No record of late 20^th^ or early 21^st^ century timber management
(6) Desired forest type (new criterion)

After scoring the entire landscape based on these six criteria, all catchment polygons (https://www.epa.gov/waterdata/nhdplus-national-data) dominated by ‘score 6’ cells were selected. We then used fire perimeter (https://frap.fire.ca.gov/mapping/gis-data/) and ESRI world imagery (https://www.arcgis.com/home/item.html?id=c03a526d94704bfb839445e80de95495) datasets to manually adjust polygon boundaries to ensure that sites primarily represented forested areas and excluded roads, infrastructure, and major rock outcrops. Final adjustments to polygon boundaries were made to meet the following criteria:

(1) Polygon area was at least 100 ha
(2) High-severity patch sizes within polygons were less than 10 ha in size
(3) Less than 10% of the polygon burned at high-severity
(4) Average of 2 or more fires within the polygon
(5) Minimal effects of edaphic conditions on forest structure (based on ESRI imagery)

From this raster scoring and polygon delineation approach, we identified a set of 119 contemporary reference sites for the Sierra Nevada yellow pine and mixed-conifer zone. Of these 119 sites, 68 sites had corresponding airborne lidar data flown at least one year after the most recent fire, while 51 sites only had structure data available from CFO. The total area covered by all reference sites was 28,556 ha, with 17,258 ha having corresponding lidar data. The minimum reference site size was 101 ha, maximum size was 966 ha, and mean size was 240 ha (Table 2).

**Table 2:** Summary count and area statistics for contemporary reference site polygons for all sites and for sites with/without corresponding airborne lidar data.

Lidar Availability	Polygon Count	Summed Area (ha)	Minimum Size (ha)	Maximum Size (ha)	Mean Size (ha)
CFO Only	51	11,298	104	966	221
Lidar Available and CFO	68	17,258	101	841	4253
**All Sites**	119	28,556	101	966	240

### 3.2 Contextualizing contemporary reference sites

Forest structural conditions in restored contemporary reference sites vary by climatic conditions across the Sierra Nevada ecoregion [3]. Thus, to assist in contextualizing the reference sites, we classified sites based on their dominant Jeronimo et al. [3] climate class. Jeronimo et al. [3] initially identified 20 climate classes for the Sierra Nevada, however only 12 of these classes were represented by our set of contemporary reference sites. In Fig. 1, we provide boxplots showing the distribution of input metrics – actual evapotranspiration (AET), climatic water deficit (CWD), and January minimum temperature (JMT) - for each of the 12 climate classes represented by the reference sites. Climate variables represent average annual values for years 1981-2010 and were downloaded from the Climate and Hydrology Basin Characterization Model website [9]. Additionally, in Fig. 2 we provide a map showing the distribution of these 12 climate classes across the Sierra Nevada region with the location of corresponding reference sites. Dominant climate class was included as a field in the contemporary reference site shapefile, and these classes were also used to organize the PDF summary document. Lastly, in Fig. 3 we show the total area of reference sites represented by each of the 12 climate classes.

### 3.3 Forest structure metrics

#### 3.3.1 Airborne lidar data

We used six airborne lidar acquisitions collected between 2018-2020 to derive a set of forest structure metrics for the contemporary reference sites. Acquisitions included North and South Plumas National Forest, Eldorado National Forest, Tuolumne County, Yosemite National Park, and the Southern Sierra All Resource Restoration (SSARR) project area. Details on flight years, total acquisition area, pulse density, and flight line overlap type are provided for each acquisition in Table 3. All data was collected during leaf-on months and met minimum pulse density and flight line overlap standards recommended for forestry-based analyses [10].

**Table 3:** Years flown, total acquisition area, mean pulse density, and average flight line overlap for each of the six lidar acquisitions used to derive forest structure metrics within the contemporary reference sites. Listed in order of priority when mosaicking.

Acquisition Name	Years Flown	Total Area (ha)	Mean Pulse Density (pulse/m^2^)	Average Flight Line Overlap
SSARR	2020	569,810	22.0	> 50%
Yosemite NP	2019	369,824	23.5	> 50%
Tuolumne County	2018/2019	694,330	15.3	> 50%
Eldorado NF	2019	577,109	28.0	> 50%
South Plumas NF	2018	560,370	12.6	> 50%
North Plumas NF	2018	466,774	13.3	> 50%

#### 3.3.2 Lidar forest structure metrics

We used the USDA Forest Service's FUSION software [11] to process all six lidar acquisitions to 1) produce a set of standard ‘gridmetrics’ and 2) apply a segmentation algorithm to identify trees from the lidar point clouds to derive additional structure metrics.

FUSION was first used to filter out non-vegetation/ground returns and to normalize all return heights using vendor-provided ground models so that Z coordinates represented vegetation height above the ground. From the normalized point clouds, we then computed a set of gridmetrics at 30-m resolution including total canopy cover, dominant canopy height, standard deviation of canopy height, canopy cover in the 2-4-m stratum, and canopy base height (Table 4). We also produced a 0.75-m resolution smoothed (using a 3 × 3 cell mean) canopy height model from which additional metrics were derived, including canopy fractal dimension index and canopy rumple index (Table 4).

In addition to the gridmetrics described above, we produced a set of metrics describing the fine-scale spatial patterns of lidar-segmented trees, which represent key reference condition metrics for historically frequent fire forests (Table 4). First, we used the watershed algorithm to segment trees from the point clouds, which we hereafter refer to as ‘tree approximate objects’ or TAOs. We then computed a set of TAO clumping metrics that describe the percentage of total TAO area occupied by various TAO clump sizes. TAOs were considered to belong to the same clump if their crowns overlapped. We also computed the percent area gap (area of each pixel not occupied by TAO crowns), percent area core gap (area of each pixel at least 6 m from TAO crowns), and TAOs per hectare within each pixel. We produced all TAO metrics at 90-m resolution since past research suggests this is approximately the scale at which fine-scale tree spatial patterns emerge in historically frequent fire forests [12].

After producing the gridmetrics, TAO-based metrics, and canopy height models for all six lidar acquisitions, we reprojected all rasters to the California Teale Albers projection (EPSG: 3310) using nearest neighbour resampling and mosaicked rasters from each metric into a single raster. For mosaicking, we prioritized acquisitions based on year flown and pulse density to 1) enable characterization of forest structure across the greatest number of sites and 2) ensure the highest quality lidar data was used for each site. Lastly, we clipped all lidar-derived structure metrics to the contemporary reference site polygons. We only provide structure metrics for reference sites in which the most recent fire occurred at least one year prior to the lidar data acquisition to account for delayed post-fire mortality.

#### 3.3.3 CFO forest structure metrics

We downloaded six forest structure metrics produced by the California Forest Observatory (CFO) representing forest conditions in year 2020 [13]. CFO structure metrics included canopy cover, canopy height, canopy base height, ladder fuel density, canopy bulk density, and canopy layer count (Table 4). We downloaded CFO rasters for all counties intersecting the Sierra Nevada ecoregion. We then mosaicked all rasters and reprojected to the California Teale Albers projection (EPSG: 3310) using nearest neighbour resampling. Lastly, we clipped the CFO forest structure rasters to the contemporary reference site polygons.

**Table 4:** Glossary of terms used in the archived datasets with common name, abbreviation, file name used in spatial datasets, and term/metric description.

Term/Metric Name	Figure Abbreviation	File Name	Description
Tree Approximate Object	TAO	NA	trees segmented from airborne lidar data using the watershed algorithm [14]
California Forest Observatory	CFO	NA	organization responsible for producing forest structure datasets for the state of California
Actual Evapotranspiration	AET	NA	“amount of water that evaporates from the surface and is transpired by plants if the total amount of water is not limited”; 30-year average from 1981-2010 [9]
Climatic Water Deficit	CWD	NA	“annual evaporative demand that exceeds available water, summed annually”; 30-year average from 1981-2010 [9]
January Minimum Temperature	JMT	NA	Minimum temperature for month of January; 30-year average from 1981-2010 [9]
Aspect	Aspect	NA	Dominant topographic aspect in radians; derived from 10-m resolution digital elevation model using R *terra* package [15]
Slope	Slope	NA	Dominant topographic slope in radians; derived from 10-m resolution digital elevation model using R *terra* package [15]
Topographic Position Index	TPI	NA	Relative elevation of 10-m resolution cell based on elevation of surrounding cells; measured within a 510-m window; low negative values represent valleys while high positive values represent ridges; derived from 10-m resolution digital elevation model using R *terra* package [15]
Canopy Cover	Canopy Cover	canopy_cover_total	all returns above 2 m divided by total number of returns; describes percentage of pixel covered by vegetation greater than 2 m in height
Dominant Canopy Height	P95 Height	p95_height	95th percentile of height values for all returns above 2 m; proxy for dominant canopy height
TAOs/ha	TAOs/ha	taos_per_hectare	number of TAOs per hectare; proxy for trees per hectare
Standard Deviation of Height	SD Height	sd_height	standard deviation of height values for all returns above 2 m; proxy for variability in tree heights
Canopy Cover 2-4 m	Cover 2-4 m	canopy_cover_2to4m_stratum	all returns within the 2- to 4-m stratum divided by all returns at or below 4 m; describes the relative canopy cover within the 2- to 4-m stratum which is a proxy for ladder fuel density
Canopy Fractal Dimension Index	FRAC Index	canopy_fractal_dimension_index	2 times the logarithm of 0.25 times the sum of the perimeter of patches of canopy all divided by the logarithm of the grid cell area; describes the degree of complexity of edges of canopy patches within each cell; lower values indicate more uniform canopy patches whereas higher values indicate more complex canopy patches
Percent Pixel Area Gap	Area Gap	percent_area_gap	percent of pixel area not covered by TAOs
Percent Canopy Single TAOs	Single TAOs	percent_single_taos	total canopy area of single TAOs divided by the total canopy area of all TAOs
Percent Canopy 2-4 TAO Clumps	2-4 Clumps	percent_2to4_tao_clumps	total canopy area of 2-4 TAO clumps divided by the total canopy area of all TAOs
Percent Canopy 5-9 TAO Clumps	5-9 Clumps	percent_5to9_tao_clumps	total canopy area of 5-9 TAO clumps divided by the total canopy area of all TAOs
Percent Canopy 10+ TAO Clumps	10+ Clumps	percent_10plus_tao_clumps	total canopy area of 10 plus TAO clumps divided by the total canopy area of all TAOs
Mean TAO Clump Size	MCS	mean_tao_clump_size	TAO clump size to which the average TAO in a pixel belongs
Canopy Base Height	NA	P25_height	25th percentile of height values for all returns above 2 m; surrogate for canopy base height
Canopy Rumple Index	NA	canopy_rumple_index	area of outer canopy surface model divided by area of underlying ground surface; describes the degree of outer canopy surface complexity; low values indicate lower canopy surface complexity whereas higher values indicate higher canopy surface complexity
Percent Area Core Gap	NA	percent_area_core_gap	precent of pixel area greater than 6 m from TAO boundaries
CFO Canopy Cover	CFO Cover	cfo_canopy_cover	“horizontal cover fraction occupied by tree canopies” [13]
CFO Canopy Height	Canopy Height	cfo_canopy_height	distance between the ground and top of the canopy [13]
CFO Canopy Bulk Density	CBD	cfo_canopy_bulk_density	“mass of available fuel that burns in a canopy fire-typically the leaves and small branches- divided by the volume of the crown” [13]
CFO Ladder Fuel Density	Ladder Fuel	cfo_ladder_fuel_density	“proportion of surface fuels in the understory”; number of returns in 1- to 4-m stratum divided by number of total returns [13]
CFO Canopy Base Height	CBH	cfo_canopy_base_height	distance between the ground and the lowest branches in the canopy [13]
CFO Canopy Layer Count	Canopy Layers	cfo_canopy_layer_count	“number of distinct vertical canopy layers” [13]

### 3.4 Forest structure distribution figures

The SNCRS_Summaries.pdf document provides summary statistics and metrics for reference sites grouped by climate class and for individual sites. Specific climate classes and associated reference sites can be accessed using the Table of Contents on page 1 of the PDF. We ordered climate classes corresponding with the order suggested in Jeronimo et al. [3], which generally ranges from lower to higher elevations and latitudes.

**Fig. 4:**
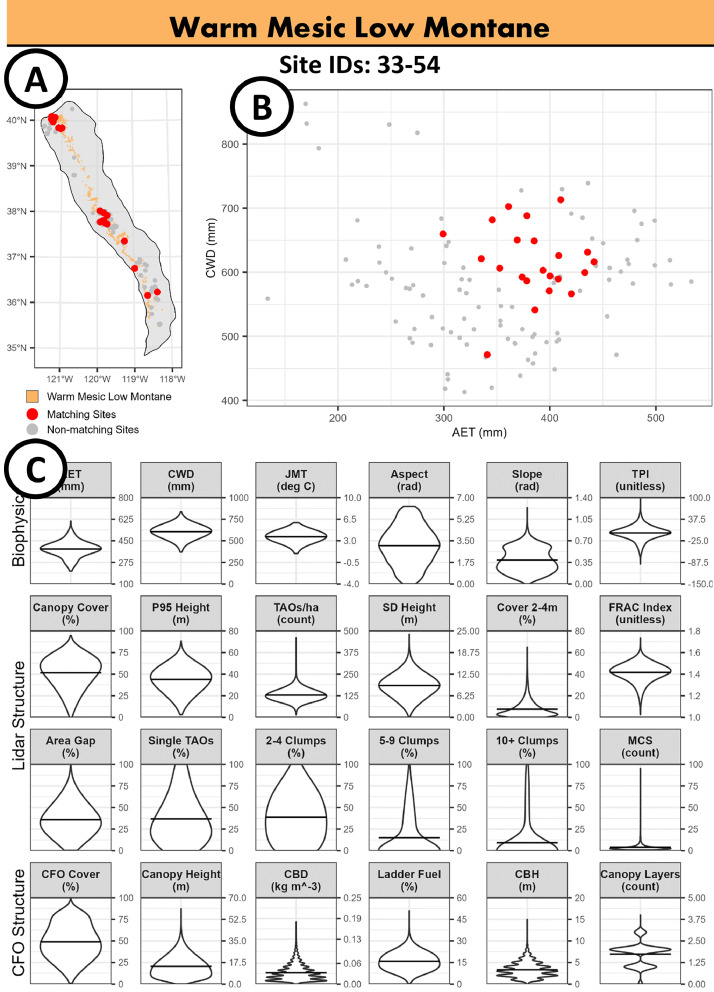
Example climate class overview page from the SNCRS_Summaries.pdf document for the Warm Mesic Low Montane climate zone [2]. Map showing the geographic location of all matching reference sites (panel A), a scatter plot showing the mean AET and CWD for each matching reference site in relation to all non-matching sites (panel B), and violin plots showing the distribution of biophysical metrics, and lidar/CFO structure metrics (panel C).

**Fig. 5:**
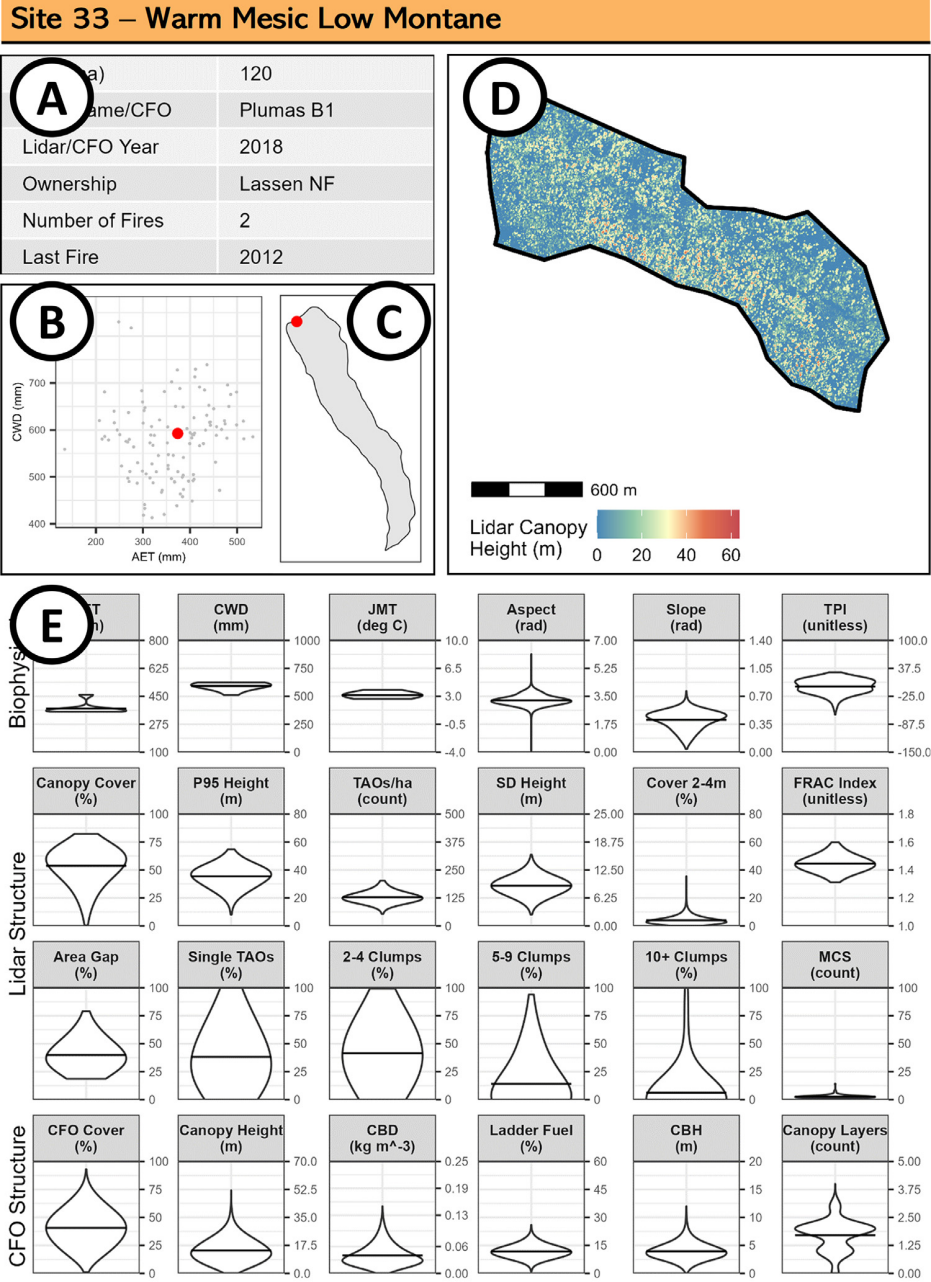
Example individual reference site overview page from the SNCRS_Summaries.pdf document for Site 33 within the Warm Mesic Low Montane climate zone [2]. Table with summary metrics and descriptions (panel A), scatterplot showing the mean AET and CWD for the given site in relation to all other sites (panel B), a map with the general geographic location (panel C), a canopy height model map derived from lidar (panel D), and violin plots showing the distribution of biophysical and lidar/CFO structure metrics (panel E).

For each of the 12 climate classes, we first summarized reference conditions across all reference sites belonging to a given class. On climate class overview pages, we provide a map showing the geographic location of all matching reference sites (Fig. 4A), a scatter plot showing the mean AET and CWD for each matching reference site in relation to all non-matching sites (Fig. 4B), and violin plots showing the distribution of biophysical metrics, lidar structure metrics (where available), and CFO structure metrics (Fig. 4C). For individual reference site overview pages, we provide a table with reference site area (ha), the name of the lidar acquisition or CFO used to derive structure metrics, the year of the lidar/CFO acquisition, the dominant ownership, the number of fires (i.e., the mean number of fires burning greater than 10% of the site), and the year of the most recent fire (Fig. 5A). We also provide a scatterplot showing the mean AET and CWD for the given site (Fig. 5B), a map with the general geographic location (Fig. 5C), a canopy height model map derived from lidar (where available) or CFO data (Fig. 5D), and violin plots showing the distribution of biophysical, lidar structure metrics (where available), and CFO structure metrics (Fig. 5E).

For all violin plots, we excluded distributions of lidar-derived canopy base height, canopy rumple index, and percent area core gap, though these metrics were provided as spatial layers in the ESRI ArcGIS Pro package. Y-axis ranges for all violin plots represent the range of a given metric across the full reference site dataset. Horizontal bars in violin plots represent the mean for a given metric. For the mean clump size metric (MCS) we truncated values at 100 to improve visualization. All violin plots bandwidths were adjusted using a multiplier of 3 for better interpretability.

## Limitations

Refer to above sections, Jeronimo et al. [3], and Chamberlain et al. [1] for detailed descriptions of limitations to primary and derived datasets.

## Ethics Statement

Datasets collected, processed, and analyzed for this publication do not require any ethics statements as suggested by Data in Brief's Guide for Authors documentation. All primary datasets used in our analyses were publicly available, did not require permission to use, and were cited where appropriate. We did not conduct any human or animal studies and no data was collected from social media sites.

## CRediT authorship contribution statement

**Caden P. Chamberlain:** Conceptualization, Methodology, Formal analysis, Writing – original draft. **Gina R. Cova:** Conceptualization, Methodology, Formal analysis, Writing – original draft. **Van R. Kane:** Conceptualization, Methodology, Writing – review & editing, Supervision, Project administration, Funding acquisition. **C. Alina Cansler:** Conceptualization, Methodology, Writing – review & editing. **Jonathan T. Kane:** Methodology, Formal analysis. **Bryce N. Bartl-Geller:** Methodology, Formal analysis. **Liz van Wagtendonk:** Conceptualization, Methodology, Writing – review & editing. **Sean M.A. Jeronimo:** Conceptualization, Methodology. **Peter Stine:** Conceptualization, Methodology, Supervision, Funding acquisition. **Malcolm P. North:** Conceptualization, Methodology.

## Data Availability

Sierra Nevada contemporary reference site boundaries and corresponding remote sensing-derived canopy structure rasters (Original data) (Forest Service Research Data Archive)
